# Intermittent Painless Hæmaturia

**Published:** 1900-02-10

**Authors:** 


[1] Brit. Med. Jour., Feb. 3.

### INTERMITTENT PAINLESS HÆMATURIA.

Cases are met with every now and then in which an intermittent hæmaturia occurs without pain, without apparent cause such as renal stone, tubercle, or cancer, and unaccompanied by any symptom by which the practitioner can localise the site of the bleeding, or ascertain its origin. By aid of the cystoscope it has been possible in some such cases to ascertain from which kidney the blood has come, and this being known, operations, such as nephrotomy, or even nephrectomy, have sometimes proved successful in its treatment. But even then our knowledge of the cause of the bleeding has remained unadvanced, for when such kidneys have been ablated and subjected to microscopic examination, nothing has been found to account for the hæmorrhage which has taken place from them. In two such cases, however, after ascertaining by cystoscopy from which kidney the blood had come, Mr. Hurry Fenwick[1] has drawn the affected kidney out upon the loin, has laid open the pelvis, and throwing into it a strong electric illumination has found the source of the bleeding to be an excessively vascular papilla. This he has removed, together with a portion of the Malpighian pyramid by means of a scoop, with the result of putting a complete stop to the hæmorrhage. Mr. Fenwick submits that the results met with in these two cases probably afford a clue to a cause for those painless unilateral hæmaturias which have so long baffled physicians by their obscurity, persistency, and danger.

